# Visual Backward masking as an endophenotype of psychosis

**DOI:** 10.1192/j.eurpsy.2023.2285

**Published:** 2023-07-19

**Authors:** M. Roinishvili, E. Chkonia, A. Brand, M. H. Herzog

**Affiliations:** 1 The University of Georgia; 2 Tbilisi State Medical University, Tbilisi, Georgia; 3 Brain Mind Institute, Swiss Federal Institute of Technology in Lausanne, Lausanne, Switzerland

## Abstract

**Introduction:**

Schizophrenia is a heterogeneous disease that is strongly influenced by genetic predisposition. A large variety of candidate genes have been identified, each of which, however, explains only a small proportion of the genetic risk for schizophrenia. Due to the complexity of psychiatric diseases, endophenotypes are of primary interest in psychiatric research. Endophenotypes are stable markers in-between the genotype and the phenotype and are thought to be associated with a small number of genes involved in the pathophysiology of the disease.

**Objectives:**

To characterize a very sensitive candidate endophenotype of schizophrenia spectrum disorders, based on visual backward masking.

**Methods:**

We tested first: Schizophrenia patients, their non-affected siblings, healthy controls and second: various populations of the schizophrenia continuum (bipolar and schizoaffective patients), as well as adolescents with psychosis, abstinent alcoholics, and depressive patients with a very sensitive masking technique.

**Results:**

Schizophrenia patients and their siblings show strong performance deficits. Masking performance of relatives was significantly in between the one of patients and controls. Moreover, deficits were stable throughout one year. The shine-through paradigm distinguishes with high sensitivity and specificity between schizophrenic patients, first-order relatives, and healthy controls. Patients with first episode of psychosis, as well as adolescents with psychosis, have shown clear performance deficit. Deficits are specific to the psychosis spectrum and not evident in depressive patients and abstinent alcoholics.

**Conclusions:**

Our results suggest that the shine-through masking paradigm is a potential endophenotype of the schizophrenia spectrum disorders.

**Disclosure of Interest:**

None Declared

